# Hospital and Institutional News

**Published:** 1921-01-15

**Authors:** 


### HOSPITAL AND INSTITUTIONAL NEWS.

### THE HOSPITAL INQUIRY.

The Parliamentary correspondent of *The Times* is responsible for the statement that Lord Cave is to be Chairman of the Committee to inquire into the position of the voluntary hospitals, and that it is hoped that the sittings will commence in about a fortnight. It will be remembered that the terms of reference were "to inquire into and report upon the financial position of the voluntary hospitals throughout the country, and to make recommendations." Dr. Addison told the House of Commons that he thought the total membership of the Committee would be five. Sir Clarendon Hyde and Mr. R. C. Norman are mentioned as possible members. We hope that the report as regards Lord Cave is correct; he is a great lawyer and a man of very great experience in affairs. The reason he has not had any close connection with hospitals is probably accidental, but the fact is fortunate, as detachment is a qualification for the office of Chairman of the Committee.

### SWOLLEN GLANDS.

Claims for the recent appearance of a so-called "new disease" do not seem, in the light of first-hand experience, to be in any sense justified. At most the slightly selective action shown towards the parotid gland by the ordinary catarrhal bacteria of the mouth and throat is relatively rare, but by no means unknown to anyone who has been a few years in medical practice. The fact that these catarrhal germs, which are ever present in practically everybody's throat, can, on occasion, attack and inflame a salivary gland, obviously having a direct connection by its duct to the mouth cavity, is by no means remarkable. The chief and only point of interest lies in the curious occurrence of yet another small outbreak of catarrhal activity in which for some reason these germs tend to provoke special effects in a particular region. Thus, at one time, numbers of people contract a "cold in the head," at another the site of general selection is the throat, but rarely does the catarrh infect haphazard the various glandular systems of the body. Nearly always the same particular set of glands is primarily effected in all the cases, and on this occasion the organism's choice is a somewhat unusual one. We have seen several detailed examinations of these recent parotid catarrhs, and, contrary apparently to general expectation, the *B. influenzae* has not been present in any of them. They appear to be simple catarrhal attacks with a rather unusual location.

### AN IMPORTANT AMALGAMATION.

The most important amalgamation of hospitals since University College absorbed the National Dental is the probable forthcoming joining of forces of the Great Northern Central Hospital and the Royal Chest Hospital, which is to be discussed at a meeting of the governors of the latter institution at the Mansion House on Monday next at 3 p.m., when the Lord Mayor will preside. The governors will be provided with a concrete scheme which has been formulated by the executives of the two hospitals. The movement arose out of the financial position of the Royal Chest Hospital, which, while not being immediately perilous, has a poor future outlook. The Minister of Health has been asked for his opinion, and, while disclaiming any jurisdiction, gives his blessing in official language to the union. Dr. Addison is interested, of course, in all public health matters, but had no statutory concern in voluntary hospitals dealing with chest complaints, except as regards tuberculosis dispensaries, and he understands that the City Road dispensary is under the scheme to be maintained. The thorny points about all amalgamations, as a rule, are chiefly in respect of the position of the medical and lay staffs, but we understand that this aspect has been made easy by the absorption of these workers in the larger organisation of the Great Northern Central. The present building in the City Road is to continue until a Royal Chest Block can be built adjoining the Great Northern Central Hospital buildings in Holloway Road. Royal Chest governors are to retain their privileges, and all memorial tablets, when the time comes, are to be removed and affixed over the new beds.

### PROFESSIONAL SECRECY.

We are glad to note that one of our medical contemporaries has expressed some not undeserved criticism of those who are so active, belated though their comments appear to be, upon the menace hidden in the new insurance medical records. It is pointed out that, as considerable public mistrust has now inevitably been caused, all those so affected should purchase for themselves, through any bookseller, the Report (Cmd. 836) of the Inter-Departmental Committee which was published last summer. At the time, curiously enough, the findings of this Committee were reported upon favourably by the general Press, and the public must remember that this Committee had been definitely appointed to decide upon the form of medical record to be employed under the terms of service for insurance practitioners in the 1920 regulations. As we pointed out last week, a new danger now lies in development of an unwarranted suspicion in the minds of the average panel patients. The Ministry have issued a clear and reassuring statement for the general public to read. We trust the patients concerned will take a considered view of the matter, for, with the *Lancet*, although we have every sympathy for and wish to support the cause of a high professional principle, we feel that statements concerning the menace to medical secrecy have this time been not a little overdone.

### AN APPEAL ARMISTICE.

Apparently Sir Ernest Hatch, Chairman of University College Hospital, thinks that there is, or should be, an armistice at the present time, in view of the forthcoming inquiry into the position of voluntary hospitals and the possible adoption of a system of concerted effort. In any case, Sir Ernest reproves the authorities of St. Thomas's Hospital in respect of their recent effort, with the kind assistance of the Lord Mayor, to do something to reduce their debt of £100,000 or more. Sir Ernest Hatch states that the inquiry which has been asked for is now in progress through the medium of the Committee promised by the Government and also by means of special movements instituted by King Edward's Fund and the British Hospitals' Association. Sir Arthur Stanley's reply is to the effect that hospitals have still got to live, even though an inquiry is progressing or promised, and, but for several other appeals before the public in the past years, St. Thomas's would have issued theirs long before this.

### THE ORGANISATION OF POST-GRADUATE MEDICINE.

The first meeting of the Executive Committee of the Fellowship of Medicine and Post-Graduate Medical Association was held on Tuesday, January 11. Amongst the matters which the General Council meeting of December 10 delegated to it for consideration and report were the advisability and practicability of appointing a general organiser of post-graduate medicine with the view of promoting facilities for post-graduate education in the United Kingdom, for which purpose it is suggested that the Government should be asked to make an annual grant. The possibility of providing a hospital solely for post-graduate study and of obtaining a grant in aid, and the provision of suitable accommodation for post-graduates, are also under consideration.

### "THE ONLY WAY."

Sir Napier Burnett is doing good work not only by his addresses, but by his writings in the lay Press. He does not neglect local newspapers. In a recent article in the *Cardigan Advertiser* he said: "The only way to deal with the [hospital] situation, to my mind, is to invest some authority with the necessary qualifications with the supervision of all hospitals throughout the country, and to allow it to allocate grants on an equitable basis. But to do this it must first have the funds available . . . . to meet the cost of repairs, *etcetra*, the amount required would be £750,000 . . . . and if a further sum of £1,000,000 could be obtained, it would be sufficient to enable all the voluntary hospitals throughout the country to overtake their arrears of work and to put them in the position which they occupied in 1914, with this difference; their annual income will still have to be increased. . . . If the welfare of the sick and suffering throughout the country is to be adequately treated, a much broader view of the hospital question will have to be taken. . . . The Joint Council of the British Red Cross Society and the Order of St. John, 'by gathering information,' will act as the great co-ordinating agency." What do the hospitals think of this solution, since decentralisation has been a cherished possession in the past, subject only to the control of the great funds, especially in London?

### TWO NEW MEDICAL KNIGHTS.

The King has been pleased to signify his intention of conferring the honour of knighthood on Maurice Craig, Esq., M.D., F.R.C.P., Physician for Psychological Medicine to Guy's Hospital, and Consulting Neurologist to the Ministry of Pensions; and also on Percival Horton-Smith Hartley, Esq., C.V.O., M.D., Physician and Lecturer in Medicine at St. Bartholomew's Hospital and Senior Physician to Brompton Hospital for Consumption.

### SOME EARLY STATISTICS.

The New Year had hardly opened when we received from the secretary of the Royal Infirmary, Leeds, full patients' statistics for 1920. The total admissions—9,480—were only fifteen less than in 1919, when many of the wards were still full of wounded soldiers, and the daily average occupation has risen from 313 to 363. We have also received the full annual report of the Royal Infirmary, Edinburgh, but there the statistical year is from October to October. In the wards during the twelve months were received 13,320 patients, and in the out-patient department 48,117. The ordinary income was £127,127, and the ordinary expenditure £130,668. A special appeal was issued during the year, and the subscriptions were increased by the substantial sum of £46,498. To effect this result a great deal of hard work must have been carried out by a large number of friends, and it is clear that the voluntary system is not languishing in the North.

### THE HISTORY OF HOSPITALS.

In connection with the last of this series of illustrated articles, which we publish on page 359, the author points out that apart from the references already given, interesting facts may be gleaned from "Walks through London," by I. C. Varrall (1815), containing a number of small fine views; Tallis's "Illustrated London" (1851), with fifteen hospital views; Sawyer's "Survey of London," published by Hogg in 1788, with three views to each page; "Londina Illustrata," an unusually fine work with rich quality in the engravings (the larger view in our third article was from this work, published by Wilkinson in 1815); Harrison's or Thornton's "History of London"; "The Picture of London" (Longman, 1818); Grose's "Antiquities"; *The Gentleman's Magazine; The Lady's Magazine*; and, for the older views, Stow's "Survey of London."

### CHANGES AT WORCESTER INFIRMARY.

By adding all sums that can at all legitimately be placed to its credit, the Worcester General Infirmary has raised some 180,000 shillings towards the fund for 200,000 shillings, started fourteen months ago. The life of every fund has its term, and this seems to have been reached in the present instance. A service of thanksgiving is to be held in the cathedral. The infirmary is losing the services of its chaplain, the Rev. G. F. Williams, whose resignation will take place in March. He has served for twenty-seven years—rather difficult financial years, at all events. Mr. Williams has now all the work of his parish on his hands, and, even were this not so, he has earned his retirement. A resolution of thanks for his services has been passed, and his successor will be appointed at the next meeting of the committee.

### THE TRAINING OF ASYLUM CHAPLAINS.

If there is always something ungrateful in the work of institutional chaplain, the task is harder than elsewhere in mental hospitals. We note with interest that the Board of Control has suggested that all clergy connected with mental hospitals should "be better equipped with a knowledge of psychology," and that courses of instruction for them should be framed in this subject. We understand that the medical superintendent of Whitchurch Mental Hospital is now discussing this proposal with the chaplains, and shall be most interested to hear the result. From the points of view of the Church and of institutions there can be little doubt that the best and most useful chaplains are those who have also received special training for their work. The missioner, to take an allied example, who is a qualified doctor as well as a priest, is usually an exceptionally able cleric, and the chaplain who has received special training benefits his prime vocation in the same way. One of the difficulties of a chaplain's position is that his duties are ill-defined, that he is supposed vaguely to be kind, and precisely to do little. The better men are apt to shun the chaplains' posts accordingly. But a proof of training for them would not only be valuable in itself, but give a new dignity to the office and its holder. We hope the suggestion above mentioned will be adopted.

### MATERNITY WARDS AND THE SICK POOR.

With the present overcrowding in London, accompanied by the extreme distress in many parts of London through the unprecedented number of unemployed, there has been a general desire to induce expectant mothers to take advantage of the facilities offered them in Poor-law infirmaries in the maternity wards. Many cases have recently come to light of people earning good money being forced to live in one or two rooms, unable to get better accommodation, and it is in these cases, where a baby is likely to be born amidst the most unsatisfactory surroundings, that Guardians are appealing to the expectant mothers to enter their well-equipped maternity wards, where they will have the best medical attention and nursing. The law of destitution is very wide, and when the present Justice Avory was legal adviser to the Local Government Board he defined destitution to embrace all those people unable to provide necessary medical and nursing accommodation at their own homes. Of course, in these cases the Guardians exercise the right to call upon the husband to contribute even up to the full cost of his wife's maintenance. Unfortunately, the Guardians have been unable to induce the powers that be to grant them facilities for separate wards for paying and non-paying patients, but if this could be done it would at once remove any prejudice that the patient might have against being in a Poor-law infirmary.

### SURGICAL SANATORIUM FOR NOTTINGHAM.

The Health Committee of the Nottingham City Council has now prepared a scheme for the provision of a sanatorium and hospital for the treatment of surgical or non-pulmonary tuberculosis. Just over a year ago the Council received a memorial from the Cripples Guild and Ransom Memorial Committee offering £6,000 towards the provision of such account. In October the Council approved plans for a sanatorium at Bulwell for pulmonary tuberculosis. As the Health Committee says, the necessity for the treatment of surgical tuberculosis is equally important and the Health Ministry is prepared to make a capital grant of £180 per head, and an annual grant of fifty per cent. of the net deficit of the Council's tuberculosis maintenance account. Hence the Committee has been in communication with the Committee of the Nottingham Cripples Guild and the Trustees of the Ransom Memorial Fund who are acting on behalf of, or in conjunction with, General Sir Joseph Laycock, who has established a sanatorium of this description at Gringley-on-the-Hill. The sanatorium consists of freehold premises which at the present time accommodate about thirty cases. General Sir Joseph Laycock has generously offered to hand over to the Corporation these premises, together with sufficient land to permit of substantial extension, with certain following reservations. Naturally the Health Committee reports that this offer should be accepted, and it has already prepared plans for enlargements, at cost of £24,000, to accommodate one hundred cases.

### A WORD TO MANCHESTER INSURANCE COMPANIES.

An interesting address was recently given in Manchester by Colonel F. H. Westmacott, and his arguments in support of the voluntary system were supported by figures, too elaborate indeed to be quoted in full, but worthy of a brief summary. Colonel Westmacott said that the total deficit of the six principal hospitals in Manchester for 1920 was £73,000. Of this sum, £35,000 concerned the Manchester Royal Infirmary, £12,000 the Children's Hospital, £8,000 Ancoats Hospital, £7,000 each the Salford Royal and St. Mary's Hospitals, and £4,000 the Royal Eye Hospital. Beside this, a total sum of £201,250 was required by these hospitals for further accommodation, increased salaries, and larger staffs. The money, he continued, could be raised only by increased subscriptions, weekly contributions in every establishment, and adequate subscriptions from the insurance companies. Very few insurance companies subscribed at all, but all should do so in return for the reduction in their liabilities secured by the voluntary hospitals' work. On the general situation Colonel Westmacott said the cost of maintaining the voluntary hospitals was seventeen millions a year. Under State or municipal control it would cost at least one-third more; the patient would become "an index-card," the rules would be rigid and administered without sympathy or discrimination, and medical education would suffer. The audience consisted of women, whom Colonel Westmacott asked to canvass every employer and to organise a hospital week each year in the city.

### MEDICAL OBITUARY.

The death took place on Saturday last of Mr. Robert Jared Bliss Howard, F.R.C.S., a surgeon who was possibly better known in a professional sense in Canada than in this country. He was the son of the late Dr. Robert Palmer Howard, of Montreal, and was educated at McGill University and the London Hospital. In 1888 he married the daughter of Lord Strathcona and Mount Royal, afterwards High Commissioner for Canada; this lady became Baroness Strathcona at her father's death. There were three sons of this marriage, one of whom was killed in action in 1915, and two daughters, one of whom was married to Lord Congleton in 1918. We much regret, also, to refer to the tragic death of Mr. Robert D. Jones, of St. Bartholomew's Hospital, who, while on a visit to his home in Wales, was knocked down by a train at a level-crossing near Penrhyn, Merionethshire, on January 7, and succumbed to his injuries on the following day.

### PAYING PATIENTS IN POOR-LAW INFIRMARIES.

On the motion of the chairman, the Rev. P. E. Mainwaring, the Newcastle Board of Guardians has passed a resolution to provide for private patients. Other Poor-law authorities are being asked, through the National Association of Poor-law Unions, to follow this example. The chairman remarked that this course would deprive Dr. Addison of his present excuse for saying that their institutions had surplus accommodation. The speaker's idea was that they should take outside patients so far as their accommodation allowed; some would be able to meet all the expenses, others only to contribute something. The alterations required would not be great, and the effect would be to safeguard their institutions from being taken over by the county borough authorities.

### A ROYAL GIFT TO BAGSHOT.

The Duke of Connaught has purchased a house in the neighbourhood of Bagshot, and intends to make the necessary alterations and additions to render it suitable as a nursing home to accommodate five in-patients and the nursing staff. It is hoped that the home, which will fill a great need in the district, will be ready for occupation next spring or early summer. In a letter received by Lady Elphinstone of Bagshot, Sir Malcolm Murray, private secretary to His Royal Highness, states that the Duke has for some time past desired to present to Bagshot a building suitable for a nursing home to perpetuate the memory of the Duchess, who spent such a great part of her life in the neighbourhood.

### THE AUCTIONS AT NORTHAMPTON.

Some interesting auctions have been held in the different wards of Northampton to relieve the General Hospital of a debt of £8,379. In spite of the severe depression in the boot trade, bidding was brisk, and some of the lots and their prices are worth recording. A 1914 sovereign fetched £2, another 22s., a half-sovereign 14s., a live pig, weighing about nine scores, 44s. per score dead weight; dead poultry realised 10s. 6d. per head, live cockerels 13s.; a reserve was placed on goods, at least in some of the auctions, and few of the lots failed to reach the price. It is thought that the sum required will have been raised, and everyone enjoyed the auctions, which were well organised in all the different wards of the borough under the spirited leadership of the Mayor, Mr. W. Harvey Reaves.

### GUARDIANS' HELP FOR WOLVERHAMPTON HOSPITAL.

The Wolverhampton Board of Guardians has unanimously adopted a report asking for powers to offer to the Committee of the General Hospital two pavilions at the New Cross Infirmary to relieve the waiting-list of 400 at the hospital. The consent of the Ministry of Health has to be sought, but so far the Guardians and the General Hospital Committee are at one. A deputation from the hospital has inspected the pavilions, which would provide 56 beds, more or less isolated, for separate and distinct use. In fact they would virtually form an annex of the hospital on Cleveland Road. The chairman of the hospital governors, Mr. T. B. Adams, is delighted at this prospect of co-operation. The patients will be probably treated by the staff of the General Hospital. The question of payment is under discussion, and the Hospital Saturday Committee has approved the scheme. We hope that it will go through and congratulate both parties on the public spirit which they have jointly shown.

### THIS WEEK'S DRUG MARKET.

The downward movement of prices being still in evidence buyers are still holding off, and do not seem to be tempted even by offers which look very much like bargains. While it is impossible to foresee how long it will be before a check is put on the general downward movement it is obvious that a more or less stable level must be reached in due course, and it is not at all improbable that when buying recommences on a considerable scale some of the articles now being offered at much reduced rates will advance in price. In any event hospital buyers would be well advised to watch quotations closely. Following on a reduction in the value of quicksilver, makers of calomel, corrosive sublimate and other salts of mercury have reduced their quotations. Citric acid has dropped considerably in price, and it is difficult to see how it can move downwards much further. Cream of tartar is also substantially lower in price. Quinine sulphate is offered at much below makers' official prices. Sandalwood oil, lemon oil, oil of cloves, lemongrass oil and citronella oil are lower in price. Most of the synthetic drugs are again cheaper, notwithstanding the possibility that the importation of these products will be regulated by a legislative measure to be introduced by the Government. Potassium bromide is again lower, and at the present price might be worth buying.

